# First National Prevalence in Italian Horse Population and Phylogenesis Highlight a Fourth Sub-Type Candidate of Equine Hepacivirus

**DOI:** 10.3390/v16040616

**Published:** 2024-04-16

**Authors:** Roberto Nardini, Giulia Pacchiarotti, Valentina Svicher, Romina Salpini, Maria Concetta Bellocchi, Raffaella Conti, Marcello Giovanni Sala, Davide La Rocca, Luca Carioti, Antonella Cersini, Giuseppe Manna, the Equine Hepatic Viruses Consortium, Maria Teresa Scicluna

**Affiliations:** 1 Istituto Zooprofilattico Sperimentale del Lazio e della Toscana “M. Aleandri”, 00178 Rome, Italy; giulia.pacchiarotti-esterno@izslt.it (G.P.); raffaella.conti@izslt.it (R.C.); marcello.sala@izslt.it (M.G.S.); davide.larocca@izslt.it (D.L.R.); antonella.cersini@izslt.it (A.C.); giuseppe.manna@izslt.it (G.M.); teresa.scicluna@izslt.it (M.T.S.); 2 Department of Biology, University of Rome Tor Vergata, 00133 Rome, Italy; valentina.svicher@uniroma2.it; 3 Department of Experimental Medicine, University of Rome Tor Vergata, 00133 Rome, Italy; romina.salpini@uniroma2.it (R.S.); bellocchi@med.uniroma2.it (M.C.B.); luca.carioti@yahoo.it (L.C.)

**Keywords:** equine hepacivirus, horses, biomolecular prevalence, Italy, phylogenesis

## Abstract

Equine hepacivirus (EqHV, *Flaviviridae*, hepacivirus) is a small, enveloped RNA virus generally causing sub-clinical hepatitis with occasional fatalities. EqHV is reported in equids worldwide, but for Italy data are limited. To address this, a survey study was set up to estimate prevalence at a national level and among different production categories (equestrian; competition; work and meat; reproduction) and national macro-regions (North, Central, South, and Islands). Data obtained testing 1801 horse serum samples by Real-Time RT PCR were compared within the categories and regions. The NS3 fragment of the PCR-positive samples was sequenced by Sanger protocol for phylogenetic and mutational analysis. The tertiary structure of the NS3 protein was also assessed. The estimated national prevalence was 4.27% [1.97–6.59, 95% CI] and no statistical differences were detected among production categories and macro-regions. The phylogenesis confirmed the distribution in Italy of the three known EqHV subtypes, also suggesting a possible fourth sub-type that, however, requires further confirmation. Mutational profiles that could also affect the NS3 binding affinity to the viral RNA were detected. The present paper demonstrates that EqHV should be included in diagnostic protocols when investigating causes of hepatitis, and in quality control protocols for blood derived products due to its parental transmission.

## 1. Introduction

The *Flaviviridae* family encompasses over 80 species of small, enveloped, single-stranded RNA viruses classified into four genera: orthoflavivirus, pestivirus, pegivirus, and hepacivirus [1,2]. These viruses are usually host-specific and mainly infect birds and mammals. Horses are susceptible to several flaviviruses, some of which are relevant zoonotic agents [1]. In the last decade, a recently discovered virus, the equine hepacivirus (hereon EqHV; family: *Flaviviridae*; genus: hepacivirus), initially described as non-primate hepacivirus in 2012 [3], then classified as hepacivirus A in 2016 [4], and recently renamed hepacivirus equi [2], gained greater attention due to its high genetic homology with the human hepatitis C virus (HCV) [3] and due to its global diffusion. As the virus is present worldwide, several studies focused on its transmission routes, which remain elusive and subject of scientific debate: to date, only parenteral transmission [5,6,7,8] and sporadic cases of vertical transmission [9,10] have been successfully demonstrated, while more data are required to evaluate the potential role of insects [11] and the role of the proximity of individuals in horizontal transmission [9,12,13,14]. Sexual transmission has not been investigated yet. Noteworthy is that the virus was also found in commercial equine products (serum and plasma) [3,4,5,6,15,16,17,18], meaning that it could evade sterilizing and control procedures, increasing the risk of its spread by veterinary therapeutic treatments [19,20]. The onset of the EqHV infection is relatively rapid: viral RNA can be detected by PCR one week post-infection in the absence of clinical signs [5,21], with a high chance of spreading unnoticed within a holding during the first stages of the infection [22]. The outcome of EqHV infection is either a sub-clinical hepatitis, which usually self-clears in a few weeks [23], or a persistent infection which could last up to a year [9,24,25,26,27]. Infected individuals usually show mild signs to none, but there are reports of poor performance, jaundice, fatigue, or discomfort in viremic subjects [14,19,24,27,28]. Moreover, correlations between EqHV infection and an increase in specific liver enzymes were assessed [3,5,7,8,21,23,24,27,28]; however, these values are also reported to frequently remain within the reference intervals or mildly increase in viremic animals [8,21,29,30]. Correlation patterns between EqHV infection and a selection of horse characteristics (age, breed, sex, production category) were also investigated. Young horses (<8 years) are often more susceptible [9,31,32,33,34,35,36] compared to older horses (>10 years) [14,37]. In thoroughbreds, a higher percentage of PCR positivity was described when compared to other breeds [15,21,31,32,33,34,38]; females seem to be more prone to infection [35,36,37] compared to males [30], and competition horses show similar higher trends of susceptibility [31,39,40,41]. These data which, however, require further verification, hint that there could be implicit management practices exposing certain individuals to a higher risk of infection. In recent years, several studies have assessed EqHV biomolecular and serological prevalence in Africa [34,36], in North America [3,20], in South America [30,35,39], in Asia [14,26,31,32,37,40,41,42,43,44], and in Australia [45]. Overall, the worldwide biomolecular EqHV prevalence ranges from <1% to 18.2% and the serological prevalence ranges from 23.9% to 83.7% (taking into account studies which include at least N ≥ 100 subjects, sampled and examined within the same time span). In Europe, EqHV prevalence has been thoroughly investigated: the first report was in 2012, in the UK [29], which highlighted a biomolecular prevalence of 2.1% (3/143); in Germany, higher biomolecular prevalence was reported, ranging from 2.4% to 18.2% [15,21,33,46,47]; in Austria, a prevalence range from 0.38% to 4.15% was reported [11]; while in France the range was from 5.6% to 6.2% [25,38]. In Italy, the only study estimating EqHV prevalence was published in 2017 [48], reporting a biomolecular prevalence of 4.7% in horses (91/1932), with the sampling performed only in two restricted Italian geographical areas: the north east and the south east, leaving out most of the peninsula and the islands.

The aims of the present study were: estimate EqHV national biomolecular prevalence in horses; verify if any statistical differences exist among the prevalences estimated for production categories (competition; equestrian; work and meat; reproduction) and macro-regions of Italy (north, center, south, and islands); perform a phylogenetic analysis on the sequences obtained from the positive samples; assess the mutation level of the analyzed sequences and also investigate their effect on the NS3 protein tertiary structure.

## 2. Materials and Methods

### 2.1. Study Design and Sampling

The sampling scheme was designed to detect an unknown prevalence (50%), with 95% Confidence Level (CL) and 5% Standard Error (SE), in the Italian horse population. Mules or donkeys were not included in the study due to difficulties in collecting samples representative of the respective populations, and due to funding limitations. The required number of samples for the parameters defined is 384 [49]. As the aims of the study also included an evaluation based on geographical and production categories, with four strata for each, the sample number was multiplied by four, resulting in 1536 samples. For convenience of stratification, 2000 samples were set for the study.

To stratify the sampling for production categories and geographical origin, thus obtaining results comparable with those of other studies, data on all the equine premises in Italy were extrapolated from the Veterinary Information System Database (https://www.vetinfo.it/). At that time, there were 12 categories, classified by ‘purpose’ and by the absence/presence of mares or stallions on the premises. To simplify this classification, the 12 categories were therefore grouped into four macro-categories, described as follows: equestrian (EQU—not competitive horses and pet horses), competition (COM—competitive horses used in sport events and races), work and meat (W/M—horses used to carry loads/work and for meat consumption), and reproduction (REP—brood mares/stallions used for breeding purposes). The macro-regions defined by the Italian National Institute of Statistics (ISTAT) are: north (Liguria, Lombardy, Piedmont, Aosta Valley, Emilia-Romagna, Friuli Venezia Giulia, Trentino South Tyrol, Veneto), center (Latium, Marche, Tuscany, Umbria), and south (Abruzzo, Basilicata, Calabria, Campania, Molise, Apulia, Sicily, Sardinia). For this study, the two islands, Sicily and Sardinia, were considered separately. The total number of samples defined in each macro-region was stratified proportionally to the total number of premises registered. Within each macro-region, the distribution in the different regions present was stratified proportionally to the number of premises registered in each region. The same stratification criteria were repeated at province level, and at this level the samples were distributed according to the proportions of the production category for each respective region. Only one sample from each premise was chosen to avoid possible bias due to repetition and holding proximity. Serum samples were randomly collected from those submitted for equine infectious anemia (EIA) surveillance activities during the 2019–2022 period by the animal health laboratory network, with which the sampling strategy was previously shared. All samples were sent to the National Reference Center for Equine Diseases (NRL-ED) and stored at −80 °C until analyzed. Upon arrival, data for each sample, as reported in the sampling form, were registered for production category and geographical position, the latter using QGIS (QGIS.org (2020–2022). QGIS Geographic Information System. Open Source Geospatial Foundation Project. http://qgis.org). When more samples than necessary were collected, the selection criteria were set as the distance among sampled premises ≥ five km.

### 2.2. Extraction and RNA Amplification

RNA extraction was performed using MagPurix^®^ EVO with the extraction kit Viral/Pathogen Nucleic Acids Extraction Kit B (Zinexts Life Science corp., New Taipei City, Taiwan), and QiaSymphony was performed with the extraction kit DSP Virus/Pathogen Mini Kit (Qiagen, Milan, Italy) according to manufacturer instructions. Products of extraction, after their analysis, were stored at −80 °C. A Real-Time RT-PCR was set up, targeting the 5′UTR fragment of the viral genome that is highly conserved; the eligible primers and probe sequences were sought through a bibliography consultation and chosen from a previously described molecular method which presented both reliability and sensitivity [3]. The selected primers are displayed in Table S1. To perform the analysis, the Luna Mastermix (Luna^®^ Universal Probe One-Step RT-qPCR Kit, New England Biolabs Inc., Ipswich, MA, USA) and the Real-Time PCR condition on QuantStudio™ 7 Flex Real-Time PCR System (Applied Biosystems^®^, Fisher Scientific Italia, Milan, Italy) were set as follows: incubation at 55 °C for 10 min, then 95 °C for 1 min, followed by 45 cycles at 95 °C for 10 s, and 60 °C for 1 min. Cycle threshold (Ct) limit was set at 40 to discriminate between PCR-positive and negative samples: samples below Ct 40 were considered positive, while samples that scored Ct ≥ 40 were either classified as negative, or repeated when results were equivocal/borderline.

### 2.3. Statistical Analysis

Prevalence was calculated as the number of positive sample against the total analyzed at national, macro-regional, and production category level. To detect potential statistical differences among the prevalence of macro-region and production categories, a Chi squared test was performed with the online tool Graphpad (https://www.graphpad.com/quickcalcs/contingency1/, accessed on 10 August 2023).

### 2.4. Phylogenetic Analysis

For the phylogenetic analysis, a conventional RT-PCR assay was set up, targeting the NS3 conserved region of the virus genome that corresponds to the protease/helicase domain of the viral polyprotein. The NS3 possesses a binding affinity to viral RNA and may be involved in viral replication activity. This region was chosen after screening the published bibliography on EqHV phylogeny studies; primers from [47] were used, considering the length of the amplicon produced (around 607 bp) and the possibility of comparing the sequenced samples with a broader online deposited dataset.

A Veriti™ 96-Well Fast Thermal Cycler (Applied Biosystems^®^, Fisher Scientific Italia, Milan, Italy) was used to perform the PCR and the SuperScript™ III One-Step RT-PCR System with Platinum™ Taq DNA Polymerase kit (Invitrogen, Applied Biosystems^®^, Fisher Scientific Italia, Milan, Italy) was employed with a primer concentration of 30 pmol/µL (0.28 µM, 0.33 µL). PCR products were visualized and verified for their length in bp in 1.5% agarose gel capillary electrophoresis using QIAxcel Advanced (Qiagen, Milan, Italy) and following manufacturer’s instructions. The samples were then recovered and purified with ExoSap kit (Applied Biosystems^®^, Fisher Scientific Italia, Milan, Italy) according to manufacturer’s instructions, and visualized again on capillary electrophoresis to ensure correct purification and the integrity of the amplicons. Sequencing was performed using Sanger Sequencing 3500 Series Genetic Analyzers (Applied Biosystems^®^, Fisher Scientific Italia, Milan, Italy) following manufacturer’s instructions. Each pair of chromatogram trace files was assembled in consensus sequence with the Tracy command line pipeline (version 0.7.3, https://bmcgenomics.biomedcentral.com/articles/10.1186/s12864-020-6635-8, accessed on 5 June 2023), which uses a progressive multiple sequence alignment algorithm (https://academic.oup.com/bioinformatics/article/25/9/1118/204548 accessed on 5 June 2023) obtaining a graph-based alignment built on pairwise overlap alignments. Sequences were edited by BioEdit Software (version 7.2.5, Tom Hall, Raleigh, NC, USA) and aligned by ClustalW Multiple Alignment internal tool. The evolutionary tree for the NS3 fragment of EqHV was inferred by using the Maximum Likelihood method, based on the Tamura–Nei model and using MEGA11 software [50]. Initial tree(s) for the heuristic search were obtained by applying the Neighbor Joining method to a matrix of pairwise distances, estimated using the Maximum Composite Likelihood (MCL) approach). In addition, the results obtained were confirmed by applying the Maximum Likelihood model produced by IQ-TREE 2 software [51], based on the TPM2+I+G4 evolutionary model chosen for the trees by ModelFinder [52]. Phylogenetic tree topology consistency was checked using the Bootstrap method, producing 1000 replicates [50]. In addition to the sequence of isolates from the present study, sequences of the EqHV NS3 gene were downloaded from Genbank. Some were taken as reference sequences (sub-type 1: JQ434002, JQ434005, JQ434008; sub-type 2, JQ434004, JQ434007; and sub-type 3: JQ434001, JQ434003, JQ434006) [3]; others and more recent sequences were used to corroborate the discrimination of these three sub-types (MK644936 [41] subt1, MT955622 [8] subt1, MZ274312 [44] subt2, MN734124 [37] subt3). Several sequences were employed to enrich the analysis with European data (MH027992, MH027996, MH028000, MH028002, MH028005 [47], KF177391 [28], JX948116 [29], KC411810, KC411812 [46]). Other Asian and American sequences were utilized to enrich the analysis with global data (LC495903, LC440467 [26], LC030422, LC030428, LC030430, LC030431, LC030432 [31], KX056116, KX056117 [32], MF152651, MF152652 (Direct Submission), NC024889 [42], KJ472766 [5], KP325403 [6]). Furthermore, some other recent Italian sequences were kindly provided by [22] (ON653391, ON653392, ON653393) and added to the analysis. Sequence KC411778 of Rodent Hepacivirus was used as outgroup [46].

### 2.5. Mutational Analysis

Sequences obtained within the setting of this study, and coding for a portion of the NS3 protein of EqHV (from aa 355 to aa 505), were analyzed using SeqScape-v2.6 software (Applied Biosystem, Thermo Fisher Scientific, Foster City, CA, USA) and were then aligned by Bioedit 7.0 software [53]. Sequences with a mixture of wild-type and mutant residues in single positions were considered to have the mutation(s) in that position. The mutation frequency for each codon of EqHV NS3 was defined as amino acid difference with respect to the EqHV NS3 reference sequence of each specific sub-type (Genbank accession number: JQ434005, JQ434004, and JQ434001 for sub-types 1, 2, and 3, respectively).

I-Tasser software [54] (https://zhanggroup.org/I-TASSER/ accessed on 5 June 2023) was used to predict the three-dimensional wt structures of Equine hepacivirus NS3 protein. The best model was aligned with the PDB model (1CU1) of the human hepacivirus (HCV) by TM-align [55], resulting in an RMSD of only 0.26 Å, supporting the robustness of the predicted structures. The structure of the EqHV NS3 protein was colored by UCSF Chimera [56].

## 3. Results

### 3.1. Study Design and Sampling

From 2019 to 2022, 1801 horse serum samples were collected and screened by Real-Time RT-PCR for the detection of EqHV RNA. The total number of samples collected exceeded the minimum number required of 1536 samples but did not reach the expected number of 2000 serums. In some cases, and in particular for the islands (Sicily and Sardinia), the distance proxy (five km distance as set on QGIS) used for the selection of the samples to be tested could not be applied because (a) the geographical distribution of the stables was non homogeneous and the majority of regional holdings were located only in a province, or in a small territory; and (b) the overall number of regional samples per category was low and the few samples received were registered regardless of the distance. In all of the above-mentioned cases, the required number of samples overruled the geographical distance, even if farms closer than one km for the same production category had not been included in the present study.

### 3.2. Real-Time PCR Results

**Figure 1:**
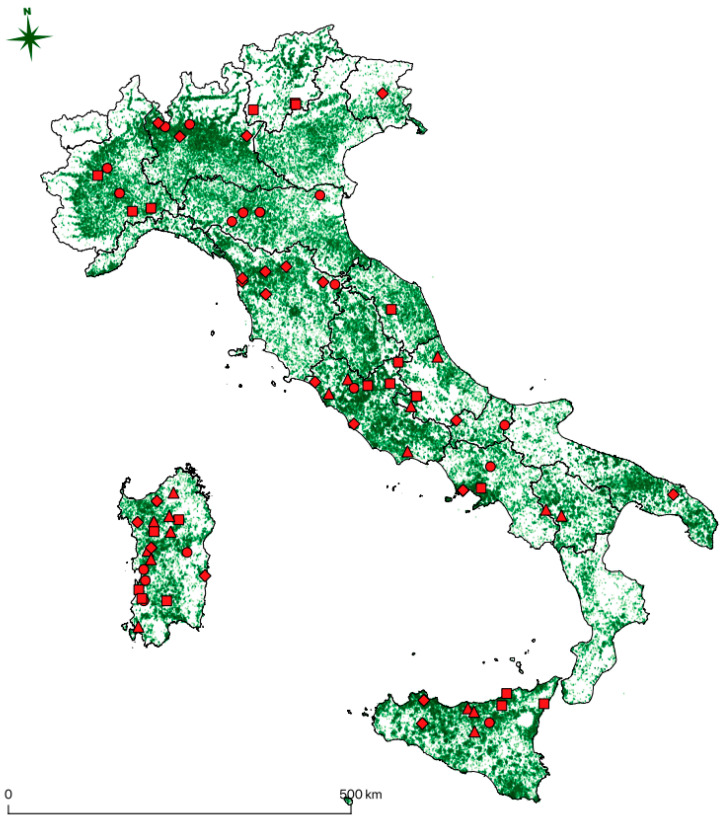
EqHV PCR-positive samples detected in this study are shown on the map. Red dots represent equestrian (EQU) positive samples, red diamonds competition (COM) positive samples, red triangles work/meat (W/M) positive samples, and red squares reproduction (REP) positive samples. Only regional borders are reported. The green shading represents the numeric density of the Italian equine premises.

The national prevalence estimated was 4.28% [1.97–6.59, 95% Confidence Interval (CI)], with 77 samples detected positive. In Table 1, total samples analyzed, total positive samples, prevalence with 95% CI, and standard error (SE) within production categories and macro-regions are presented. Geographical distributions of the analyzed samples are shown in the Supplementary information from Figures S1–S4, while that of the positive samples is shown in Figure 1.

### 3.3. Statistical Analysis

The results of the Chi Square test are displayed in Table 2 and Table 3 for macro-regions and production categories, respectively. No statistically significant differences were identified among the prevalences of the four production categories and five macro-regions.

### 3.4. Phylogenetic Analysis

**Figure 2:**
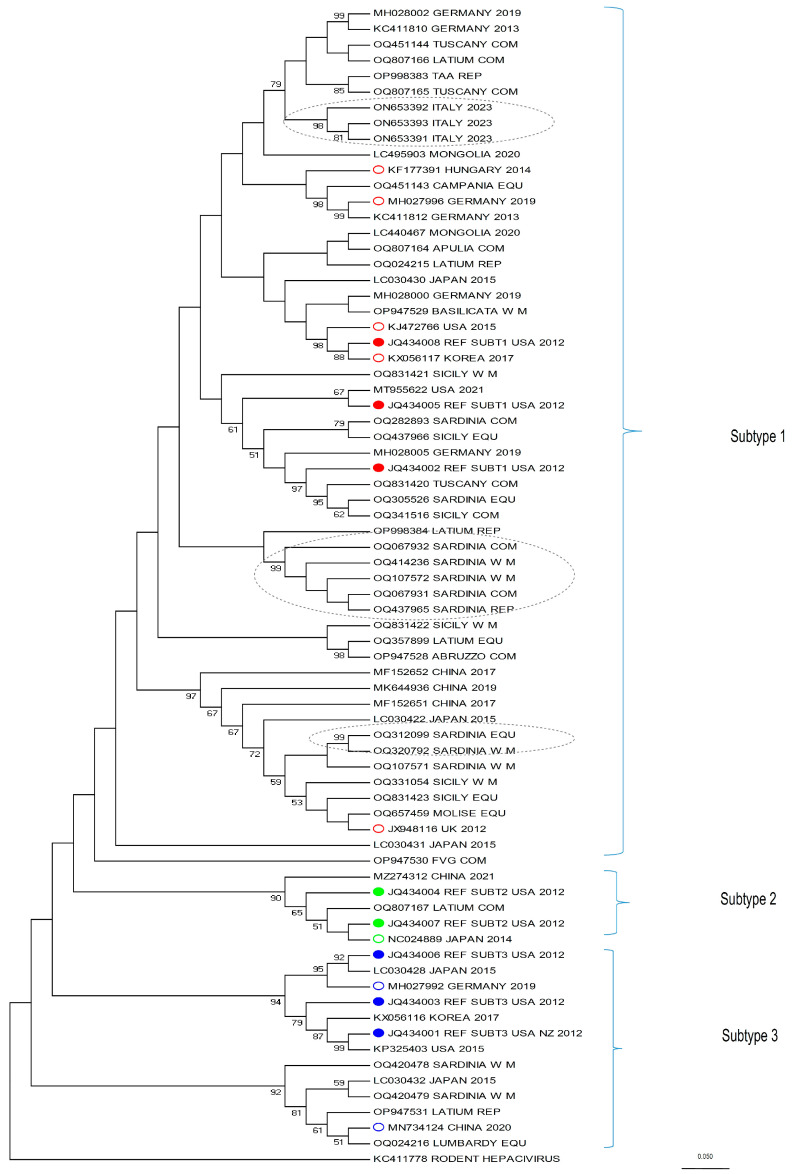
Phylogenetic analysis of NS3 partial gene of equine hepacivirus. The figure reports the phylogenetic tree based on 497-nt fragment of NS3 gene of 35 EqHV strains detected in the present study or retrieved from GenBank database, including the reference sequences of sub-types 1 (O and ●), 2 (O and ●), and 3 (O and ●). Molecular evolutionary analyses were performed using Maximum Likelihood method based on the Tamura–Nei model with bootstrap test (n = 1000). Bootstrap values > 50% are shown. In grey dotted ellipses, the potential transmission clusters can be observed (all supported by 99% of bootstrap). Sequence KC411778 [46] of rodent hepacivirus was used as outgroup. Scale bar indicates nt substitutions per site.

Among the 77 positive samples, the NS3 sequence was obtained for 35 (length: 497 bp). Phylogenetic analysis was performed using the Maximum Likelihood method, including the reference sequences of sub-types 1, 2, and 3, available in literature [3], to assign a sub-type to each of the sequences. The phylogenetic tree with the highest log likelihood (−6324.5024) is shown in Figure 2. The percentage of trees in which the associated taxa clustered together is shown next to the branches. 

The majority of the sequences were classified as sub-type 1 (30/35, 85.7%), followed by sub-type 3 (4/35, 11.4%), and sub-type 2 (1/35, 2.9%). Notably, two potential transmission clusters were observed (all supported by 99% of bootstrap): one composed of five samples and the second of two samples. Both clusters included viral strains infecting animals from the island of Sardinia. A group of sequences seem to cluster in a fourth sub-type that was never reported.

### 3.5. Mutational Analysis

**Figure 3:**
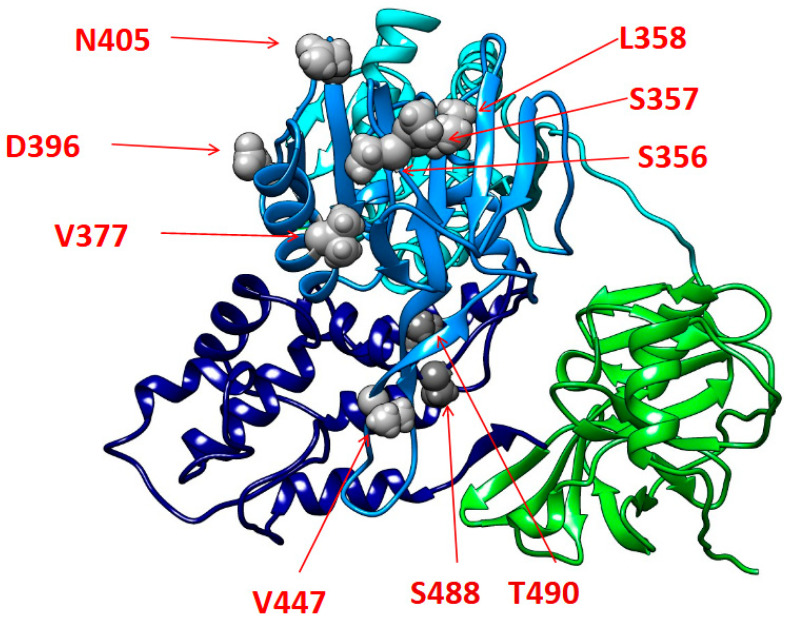
The three-dimensional structure of equine hepacivirus NS3 protein constructed by I-Tasser software using the PDB model of the human hepacivirus (HCV 1CU1) as reference. The functional domains are color-coded in the equine hepacivirus NS3 protein as follows: serine protease domain (1–180, green); helicase subdomain I (181–326 cyan); helicase subdomain II (327–481 dodger blue); helicase subdomain III (482–631 navy blue); residues S356, S357, L358, V377, D396, N405, V447, S488, and T490, showing their side chains as spheres, are reported in grey.

The mutational analysis of the EqHV NS3 serine protease/helicase (aa 348–505) revealed the presence of at least one specific mutation, differentiating the viral isolates of our study from the reference sequence of each sub-type in almost all EqHV-infected subjects (94.3%). In particular, complex mutational patterns, characterized by the co-presence of >2 NS3 mutations, were identified in EqHV strains detected in 11 horses (31.4%). In Table 4 and Table 5, prevalences of detected mutations are displayed, stratified according to production categories and macro-regions, respectively. Notably, the mutation occurring with the highest frequency among the analyzed viral sequences was T490V (77.1%). Notably, this mutation was identified in EqHV sequences derived from almost all Italian regions, indicating that it is the dominant viral species across Italy. Moreover, L358I was found in 20% of the overall analyzed EqHV strains, having been detected in several Italian regions (Latium, Lombardy, Sardinia, and Sicily), thus highlighting its relevant distribution across our country. Similarly, V377I and V447I were identified in viral strains from two different regions with an overall prevalence of 8.6% and 5.7%, respectively. Lastly, additional mutations with a peculiar geographic-dependent distribution were also present. In particular, in the present defined sample, S488T and N405K were found to circulate exclusively in EqHV-infected horses in Sardinia, with a prevalence of 14.3% and 8.6%, respectively. Lastly, four other mutations (S356A, S357A, T358L, D396E) were detected altogether in a single subject. Most of the identified mutations reside within alfa-helixes of the subdomains II and III of the NS3 helicase functional region, as shown in Figure 3 and Table 6.

## 4. Discussion

Here we present the first study on EqHV prevalence at a national level, with the aim of providing further data regarding the distribution of this virus in Italy, both geographically and among horse production categories. In addition, we present the results of the phylogenetic and the NS3 tertiary structure analysis.

The study presents some limitations: although it was designed as a cross-sectional study, the COVID-19 pandemic in 2020 brought activities almost to a complete halt. This obliged us to further extend the sampling time for over a year and a half to reach the required sample size. Thus, the prevalence data presented cannot be considered as punctual, but instead refer to a time span. Furthermore, the study was designed to proportionally sample the horse population, reflecting its distribution on Italian territory, in order to estimate an accurate EqHV biomolecular prevalence in Italian horse production categories between 2019–2022. However, the collection of samples from some provinces and regions was harder during and after the COVID-19 pandemic; nevertheless, the sampling level achieved was considered reliable since the SE resulted in <5%, as set in the study design. Slight SE variances were observed in the macro-region comparison, but all values were close to the expected one. Another limitation of the present study was that data on the age, breed, and sex of the analyzed horses were not collected and therefore no retrospective comparisons, nor statistical analysis could be inferred regarding the influence that these risk factors could have played in the study scenario. Investigating these variables would require a greater number of samples that was not achievable with the funding resources available for the project. For the same reason, prevalence in donkeys and mules was not investigated, and priority was given to horses considering that they are the species most present and with the highest economic value.

However, despite these limitations, this study presents the first datum of EqHV biomolecular prevalence at national level in Italian horses. The described prevalence of 4.27% is coherent with what was previously reported in Italy (4.7%) [48] and within the range of biomolecular prevalence reported both in Europe (<1–18.2%) [11,15,21,25,29,33,38,46,47,57,58] and worldwide (3.2–46.2%) [3,14,20,26,30,31,32,34,35,36,37,39,40,41,42,43,44,45].

It should be pointed out that higher biomolecular prevalence (40–46.2%) is described in the literature [22,26,33,41] when the sample is collected within the same holdings; on the contrary, lower prevalence (3.4–5.6%) is described when the sampling was set to assess the prevalence in a geographic area [11,25,40,48]. Intra-premises prevalence appears higher worldwide; in fact, once introduced in a stable, the virus seems to spread rapidly with a still unclear transmission route and, because of its asymptomatic development, it is feasible that one or more carriers can infect other horses without showing any evident clinical signs.

The sampling was designed to also investigate four horse production categories: equestrian, competition, work/meat, and reproduction, which reflect the different categories within which holdings are officially classified by the Italian animal health authorities. This categorization was established to study whether different management practices could play a role in the spread of the virus. As a matter of fact, the literature reports that competition horses and breeding horses are often more susceptible to infection than other categories [31,35,36,37,39,40,41]. In this study, the majority of PCR-positive samples were grouped within the competition and reproduction categories, although statistical analysis did not highlight any significant difference among the production categories. Further studies are necessary to confirm these results with a higher number of samples.

Nonetheless, the data collected in this study can be useful to highlight and confirm that EqHV infection poses a potential threat to animal health, being spread across all categories, and could have a tangible impact on the sport industry, which has the highest economic turnover. In fact, poor performances, lethargy, apathy and fatigue are widely reported [14,19,24,27,28,33], with occasional cases of euthanasia due to poor overall health conditions and severe hepatic damage [19,21,24]. Data on EqHV biomolecular prevalence also highlight another issue related to therapies using blood, plasma, and other blood derivatives that are commercially available, as well as blood transfusions between horses. EqHV was already detected in some commercial blood products [15,16,17,18], and therefore it is crucial to amend quality control analysis to certify the commercial products as EqHV-free (together with the exclusion of other etiological agents that can be relevant for equine health), and to include this virus in veterinary diagnostic protocols to control its spread in the equine population via non-commercial transfusions.

At the present time there is no therapy available for EqHV, thus biosecurity measures to reduce the spread of the disease on a holding and between holdings are as follows: isolate the suspect case(s), suspend any production of blood products (if on a holding with this purpose), and periodically control the animals to monitor for the presence of the virus. Infection may last for a period of six months [5,6,8] after which the subject recovers, and no natural cases of resurgence of infection are reported so far, although re-infection has been reported [7,8]. On the contrary, if the virus can still be detected after six months, the subject is considered chronically infected [5,6,24,26,27,28,59] and should be managed with caution.

This study also provides comprehensive data on the phylogenetic characteristics of the isolates from the whole Italian territory, integrating the already available data that were limited to some regions only. For this purpose, we analyzed a portion of the NS3 fragment, which is the serine protease/helicase domain of the viral polyprotein. Although EqHV is an RNA virus, and therefore prone to mutation, the strains of the present study mostly belong to the three known EqHV sub-types (Figure 2). As expected from the literature [30,37,44], the majority of the sequences (30) clustered within sub-type 1, while four were identified as sub-type 3 and only one sequence as sub-type 2.

Some clusters in sub-type 1 (identified in Figure 2 with grey dotted ellipses) are of particular interest. From top to bottom, the first cluster is represented by three very recent sequences from an Italian EqHV outbreak (kindly provided by the authors of [22]), which are relevant because the authors could follow up the infection of a few horses for several months, allowing for the tracking of viral mutation within the same subjects. This is the case for ON653391 and ON653393, detected from the same horse sampled six months apart, which clearly shows that EqHV is prone to mutations (for more in-depth considerations, see [22]). The second and third clusters, instead, include sequences from the present study and are located mid-way and at the bottom of sub-type 1, respectively. The first one includes five sequences from Sardinia (OQ067932, OQ414236, OQ107572, OQ067931, OQ437965) and the second includes two sequences, also from Sardinia (OQ312099 and OQ320792). Both clusters show a 99 bootstrap value. A retrospective tracing of the subjects allowed us to determine that (a) the premises of origin are distant from each other (>12 km to <250 km), apart from two samples which do not belong to the same cluster, nor to the same production category, but for which the stables of origin are located 2.1 km apart (Accession Number ID: OQ067932; OQ320792); (b) being geographically distributed, they could hardly share the same veterinarian, except from the aforementioned two subjects; (c) none of the seven horses had a history of movement to or from the other stables, according to the data available on the Veterinary Information System Online Database; (d) data on veterinary treatments (such as vaccines, plasma transfusion, etc.) could not be retrieved. The reason why these sequences cluster so closely together, then, cannot be assessed confidently, but it must be considered that each stable held a discrete number of horses (>1 to <21) at the time of sampling, therefore it cannot be ruled out that the movement of an EqHV-positive horse, different from those sampled, could be the source of infection for each respective cluster.

Almost all of the positive subjects detected in this study, as reported in Figure 1, live in territory with a high density of equine premises, thus representing a threat for the spread of the virus through movement or other unknown transmission routes. Movements from one stable to another are indeed a common practice in the horse industry, either for trade, management, breeding, or sport activities. Since the virus appears widespread and the sequences are phylogenetically close regardless of their origin, presumably movements have played a role in the spread of the three sub-types worldwide in the last decade, and probably before that. When it comes to horse pre-movement sanitary controls, several diseases are screened, but EqHV is not currently present in routine laboratory diagnosis, at least in Italy, and probably also in most countries of the world. As preventive measures, stricter diagnostic protocols when moving animals should be considered, especially when susceptible breeds or valuable individuals are involved.

Further, regarding the phylogenetic analysis, another interesting aspect of the tree should be discussed: included in sub-type 3 there is a branch (the second branch bottom-up, identified by a 92 bootstrap value) which clusters alone and originates from a different node when compared to the other branches of the tree. Four sequences from the present study are included in this group (OQ420478, OQ420479, OP947531, OQ024216) along with two sequences retrieved from Genbank (LC030432 [31] and MN734124 [37]). Other recent phylogenetic analyses [22,26,37,44,60] and the original paper from [31], show a similar outcome. In these papers, in fact, the branch, identified either by LC030432 [31], MN734124 [37], or both, separates from the node which includes the sequences used as reference for sub-type 3 (JQ434001, JQ434003, JQ434006 [3]). This suggests that these sequences could substantially differ from those included in sub-type 3 and from the other sub-types; it could be hypothesized that this branch was never part of EqHV sub-type 3, but it represents a totally different sub-type which was until now under-represented and that was therefore considered part of sub-type 3.

The most accredited criteria supporting the proposal of new sub-types for EqHV are those described in the literature for HCV [4,61,62], which were already used to introduce the presence of sub-type 2 [30,38] and 3 [41] for EqHV. These criteria identify the need for specific parameters to assess the detection of a new sub-type, such as a “complete or nearly complete coding region sequence differing from other sequences by at least 15% of nucleotide positions” and “sequence information from at least two other isolates in core/E1 […] and NS5B” [62], which could not be fulfilled in the present study mainly for funding reasons. However, a larger set of sequences, representative of this branch and that could be used as reference, are those of the NS3 protein submitted by [31] (LC030420, LC030425, LC030426, LC030427, LC030432), the complete polyprotein sequence by [37] (MN734124), and the new partial NS3 sequences from the present study (OQ420478, OQ420479, OP947531, OQ024216). We are strongly looking forward to further studies and broader sampling from horses worldwide to verify this hypothesis that could update the classification of EqHV.

Regarding the mutational analysis, most of the identified mutations reside within the alfa-helixes of the II and III subdomains of the NS3 helicase functional regions. In particular, these mutations are localized in portions that are critical for the RNA binding and the following unwinding activity of NS3. In this regard, previous studies, in reference to the homologous NS3 protein of the human hepatitis C virus, have shown that amino acid substitutions in NS3 C-terminal alfa-helixes can influence RNA replication with a beneficial impact on the variant’s replicative fitness [63]. On this basis, it could be speculated that the reported mutations may play a similar role, with a potential enhancing impact on EqHV replicative capability. However, further in-depth in vitro studies are necessary to confirm how the mutations accumulated in these regions could impact on NS3 proper folding and its binding affinity to viral RNA, and, in turn, to finally elucidate their potential implications on the viral replication activity. Lastly, a more extensive and comprehensive mutational analysis of full-length EqHV sequences will be critical to better characterize the mutational profiles of EqHV strains circulating in Italy.

## 5. Conclusions

Equine hepacivirus is a recently discovered virus of equids. It affects the liver of infected horses and causes sub-clinical hepatitis, seldom leading to death. Its presence is demonstrated worldwide and, since the virus has also been detected in blood products, it represents a threat for animal health in veterinary practice. The present study reports the first data on national prevalence in Italy, demonstrating its circulation at national level. Phylogenetic analysis shows that the Italian strains connect with isolates present worldwide, and thus the extensive circulation of the virus is confirmed. Moreover, the possible presence of a fourth sub-type is discussed, but further studies are needed to verify this hypothesis. All considered, EqHV remains a relevant virus in the equine infectious disease landscape and should be addressed with care, highlighting all the more its importance in diagnostic protocols for hepatitis in horses and in blood product safety control tests.

## Figures and Tables

**Table 1:** Total analyzed samples, total positive samples, prevalence with 95% CI, and SE values within categories (upper table) and macro-region (lower table).

	Analyzed Samples	Positive Samples	P (%)	Lower IC (%)	Upper IC (%)	SE (%)
**Production Categories**						
Equestrian	497	17	3.42	0	7.82	±4.40
Competition	464	23	4.96	0.41	9.51	±4.55
Work/Meat	442	17	3.85	0	8.51	±4.66
Reproduction	398	20	5.03	0.11	9.94	±4.91
**Italy Macro-Regions**						
North	393	18	4.58	0	9.52	±4.94
Center	315	17	5.40	0	10.92	±5.52
South	435	13	2.99	0	7.96	±4.70
Sardinia	376	20	5.32	0.26	10.37	±5.05
Sicily	282	9	3.19	0	9.03	±5.84

**Table 2:** Chi Square p-values of the pair comparison among production categories.

Chi Square P	Reproduction	Work/Meat	Competition
**Equestrian**	0.23	0.72	0.23
**Competition**	0.96	0.41	
**Work/Meat**	0.40		

**Table 3:** Chi Square p-values of the pair comparison among macro-regions.

Chi Square P	Sicily	Sardinia	South	Center
**North**	0.43	0.740	0.272	0.73
**Center**	0.23	1.00	0.130	
**South**	1.00	0.11		
**Sardinia**	0.25			

**Table 4:** Prevalence of NS3 mutations in the overall study population (T = 35) stratified according to the different production category. T = total sequences for each column; N = number of sequence showing the mutation indicated in the row heading; (%) = percentage of sequence with the given mutation in relation to the total.

NS3 Mutation	Overall, N (%)	Competition, N (%) T = 12	Equestrian, N (%) T = 8	Reproduction, N (%), T = 5	Work/Meat, N (%) T = 10
T490V	27 (77.1)	9 (75)	6 (75)	4 (80)	8 (80)
L358I	7 (20.0)	1 (8.3)	2 (25)	1 (20)	3 (30)
S488T	5 (14.3)	2 (16.7)	0 (0)	1 (20)	2 (20)
V377I	3 (8.6)	0 (0)	0 (0)	0 (0)	3 (30)
N405K	2 (5.7)	1 (8.3)	0 (0)	1 (20)	0 (0)
V447I	2 (5.7)	1 (8.3)	1 (12.5)	0 (0)	0 (0)
D396E	1 (2.9)	1 (8.3)	0 (0)	0 (0)	0 (0)
S356A	1 (2.9)	1 (8.3)	0 (0)	0 (0)	0 (0)
S357A	1 (2.9)	1 (8.3)	0 (0)	0 (0)	0 (0)
T358L	1 (2.9)	1 (8.3)	0 (0)	0 (0)	0 (0)

**Table 5:** Prevalence of NS3 mutations in the overall study population (N = 35) stratified according to geographical area: (a) Central Italy includes 9 EqHV strains isolated from Latium (N = 6) and Tuscany (N = 3); (b) Southern Italy includes 5 EqHV strains isolated from Apulia, Basilicata, Molise, Campania, and Abruzzi (N = 1 for each region); (c) Northern Italy includes 3 EqHV strains isolated from Lombardy, Friuli Venezia Giulia, and Trentino Alto Adige (N = 1 for each region).

NS3 Mutations	Overall, N (%) N = 35	Sardinia, N (%) (N = 12)	Sicily, N (%) (N = 6)	Central Italy ^a^, N (%) (N = 9)	Southern Italy ^b^, N (%) (N = 5)	Northern Italy ^c^, N (%) (N = 3)
T490V	27 (77.1)	10 (83.3)	4 (66.7)	6 (66.7)	5 (100)	2 (66.7)
L358I	7 (20.0)	4 (33.3)	1 (16.7)	1 (11.1)	0 (0)	1 (33.3)
S488T	5 (14.3)	5 (41.7)	0 (0)	0 (0)	0 (0)	0 (0)
V377I	3 (8.6)	2 (16.7)	1 (16.7)	0 (0)	0 (0)	0 (0)
N405K	2 (5.7)	2 (16.7)	0 (0)	0 (0)	0 (0)	0 (0)
V447I	2 (5.7)	0 (0)	1 (16.7)	1 (11.1)	0 (0)	0 (0)
D396E	1 (2.9)	1 (8.3)	0 (0)	0 (0)	0 (0)	0 (0)
S356A	1 (2.9)	0 (0)	0 (0)	1 (11.1)	0 (0)	0 (0)
S357A	1 (2.9)	0 (0)	0 (0)	1 (11.1)	0 (0)	0 (0)
T358L	1 (2.9)	0 (0)	0 (0)	1 (11.1)	0 (0)	0 (0)

**Table 6:** Localization of the identified mutations within the NS3 domains.

NS3 Mutations	Localization as Secondary Structure	NS3 Functional Domain
S356A	Helix 356-358	Helicase subdomain II
S357A	Helix 356-358	Helicase subdomain II
T358L	Helix 356-358	Helicase subdomain II
L358I	Helix 356-358	Helicase subdomain II
V377I	Helix 371-384	Helicase subdomain II
D396E	Coil 392-405	Helicase subdomain II
N405K	Strand 405-410	Helicase subdomain II
V447I	Helix 447-502	Helicase subdomain II
S488T	Helix 488-500	Helicase subdomain III
T490V	Helix 488-500	Helicase subdomain III

## Data Availability

The data that support the findings of this study are available from the corresponding author, R.N., upon reasonable request.

## Supplementary Materials

The following supporting information can be downloaded at: https://www.mdpi.com/article/10.3390/v16040616/s1, Equine Hepatic Viruses Consortium (alphabetical order). Figure S1: Collection sites of the serum samples belonging to the Equestrian (EQ) category. Figure S2: Collection sites of the serum samples belonging to the Competition (COMP) category. Figure S3: Collection sites of the serum samples belonging to the Work/Meat (W/M) category. Figure S4: Collection sites of the serum samples belonging to the Reproduction (REP) category: Table S1: Primers and probe used for the RT-Real time PCR and the Endpoint PCR.
