# Supplementary material for: First National Prevalence in Italian Horse Population and Phylogenesis Highlight a Fourth Sub-Type Candidate of Equine Hepacivirus
Source: Viruses. 2024 Apr 16;16(4):616. doi: 10.3390/v16040616 (PMC11054338; doi:10.3390/v16040616)
Supplement: Supplementary file 1 [file viruses-16-00616-s001.zip › viruses-2946002-supplementary.pdf]

Supporting Information for

First national prevalence in Italian horse population and phylogenesis highlights a fourth sub-type candidate of Equine Hepacivirus

This PDF file includes:

- Equine Hepatic Viruses Consortium (alphabetical order)
- Figures S1 to S4
- Table S1

Equine Hepatic Viruses Consortium (alphabetical order)

Giuseppe Addis: Istituto Zooprofilattico Sperimentale della Sardegna, 07100 Sassari, Italy, giuseppe.addis@izs-sardegna.it

Sara Andreatta: Istituto Zooprofilattico Sperimentale delle Venezie, 35020 Legnaro, PD, Italy, sandreatta@izsvenezie.it

Marco Bregoli: Istituto Zooprofilattico Sperimentale delle Venezie, 35020 Legnaro, PD, Italy, mbregoli@izsvenezie.it

Giuseppina Brocherel: Istituto Zooprofilattico Sperimentale del Lazio e della Toscana "M. Aleandri", 00178 Rome, Italy, giuseppina.brocherel@izslt.it

Diego Brundu: Istituto Zooprofilattico Sperimentale della Sardegna, 07100 Sassari, Italy, diego.brundu@izs-sardegna.it

Gianpaolo Bruni: Istituto Zooprofilattico Sperimentale del Lazio e della Toscana "M. Aleandri", 00178 Rome, Italy, gianpaolo.bruni@izslt.it

Antonio Cacia: Istituto Zooprofilattico Sperimentale del Mezzogiorno, Portici, Italy, antonio.cacia@izsmportici.it

Roberta Catanzariti: Istituto Zooprofilattico Sperimentale della Puglia e della Basilicata, Foggia, Italy, roberta.catanzariti@izspb.it

Nicola Cavaliere: Istituto Zooprofilattico Sperimentale della Puglia e della Basilicata, Foggia, Italy, nicola.cavaliere@izspb.it

Stefano Colorio: Istituto Zooprofilattico Sperimentale delle Venezie, 35020 Legnaro, PD, Italy, scolorio@izsvenezie.it

Berardina Costantini: Istituto Zooprofilattico Sperimentale dell'Abruzzo e del Molise, Campo Boario, 64100 Teramo, Italy, b.costantini@izs.it

Silva Costarelli: Istituto Zooprofilattico Sperimentale dell'Umbria e delle Marche "Togo Rosati", Via Salvemini 1, 06126 Perugia, Italy, s.costarelli@izsum.it

Antonella De Angelis: Istituto Zooprofilattico Sperimentale del Mezzogiorno, Portici, Italy, antonella.deangelis@izsmportici.it

Claudio De Martinis: Istituto Zooprofilattico Sperimentale del Mezzogiorno, Portici, Italy, claudio.demartinis@izsmportici.it

Monica Dellepiane: Experimental Zooprophylactic Institute of Piedmont, Liguria and Aosta Valley (IZSPLV), Via Bologna 148, 10154 Turin, Italy, monica.dellepiane@izsto.it

Chiara Di Pancrazio: Istituto Zooprofilattico Sperimentale dell'Abruzzo e del Molise, Campo Boario, 64100 Teramo, Italy, c.dipancrazio@izs.it

Anna Duranti: Istituto Zooprofilattico Sperimentale dell'Umbria e delle Marche "Togo Rosati", Via Salvemini 1, 06126 Perugia, Italy, a.duranti@izsum.it

Giovanni Farina: Istituto Zooprofilattico Sperimentale delle Venezie, 35020 Legnaro, PD, Italy, gfarina@izsvenezie.it

Giorgio Fezia: Experimental Zooprophylactic Institute of Piedmont, Liguria and Aosta Valley (IZSPLV), Via Bologna 148, 10154 Turin, Italy, giorgio.fezia@izsto.it

Giovanna Fusco: Istituto Zooprofilattico Sperimentale del Mezzogiorno, Portici, Italy, giovanna.fusco@izsmportici.it

Stefano Gavaudan: Istituto Zooprofilattico Sperimentale dell'Umbria e delle Marche "Togo Rosati", Via Salvemini 1, 06126 Perugia, Italy, s.gavaudan@izsum.it

Federica Gobbo: Istituto Zooprofilattico Sperimentale delle Venezie, 35020 Legnaro, PD, Italy, fgobbo@izsvenezie.it

Barbara Grossele: Istituto Zooprofilattico Sperimentale delle Venezie, 35020 Legnaro, PD, Italy, bgrossele@izsvenezie.it

Francesca Gucciardi: Istituto Zooprofilattico Sperimentale della Sicilia "A. Mirri", Via Marinuzzi, 90129 Palermo, Italy, francesca.gucciardi@izssicilia.it

Annalisa Guercio: Istituto Zooprofilattico Sperimentale della Sicilia "A. Mirri", Via Marinuzzi, 90129 Palermo, Italy, annalisa.guercio63@gmail.com

Giulia Maioli: Istituto Zooprofilattico Sperimentale della Lombardia e dell'Emilia Romagna "Bruno Ubertini" (IZSLER), Via Antonio Bianchi 7/9, 25124 Brescia, Italy, giulia.maioli@izsler.it

Maria Lucia Mandola: Experimental Zooprophylactic Institute of Piedmont, Liguria and Aosta Valley (IZSPLV), Via Bologna 148, 10154 Turin, Italy, marialucia.mandola@izsto.it

Lucia Palazzo: Istituto Zooprofilattico Sperimentale della Puglia e della Basilicata, Foggia, Italy, lucia.palazzo@izspb.it

Marta Paniccià: Istituto Zooprofilattico Sperimentale dell'Umbria e delle Marche "Togo Rosati", Via Salvemini 1, 06126 Perugia, Italy, m.paniccia@izsum.it

Antonio Parisi: Istituto Zooprofilattico Sperimentale della Puglia e della Basilicata, Foggia, Italy, antonio.parisi@izspb.it

Aureliana Pedditzi: Istituto Zooprofilattico Sperimentale della Sardegna, 07100 Sassari, Italy, aureliana.pedditzi@izs-sardegna.it

Giuseppa Purpari: Istituto Zooprofilattico Sperimentale della Sicilia "A. Mirri", Via Marinuzzi, 90129 Palermo, Italy, giuseppa.purpari@izssicilia.it

Maria Renzi: Istituto Zooprofilattico Sperimentale della Lombardia e dell'Emilia Romagna "Bruno Ubertini" (IZSLER), Via Antonio Bianchi 7/9, 25124 Brescia, Italy, maria.renzi@izsler.it

Addolorato Ruberto: Istituto Zooprofilattico Sperimentale dell'Abruzzo e del Molise, Campo Boario, 64100 Teramo, Italy, a.ruberto@izs.it

Angelo Ruiu: Istituto Zooprofilattico Sperimentale della Sardegna, 07100 Sassari, Italy, angelo.ruiu@izs-sardegna.it

Giovanni Sala: Istituto Zooprofilattico Sperimentale della Lombardia e dell'Emilia Romagna "Bruno Ubertini" (IZSLER), Via Antonio Bianchi 7/9, 25124 Brescia, Italy, giovanni.sala@izsler.it

Erminia Sezzi: Istituto Zooprofilattico Sperimentale del Lazio e della Toscana "M. Aleandri", 00178 Rome, Italy, erminia.sezzi@izslt.it

Alessandra Stancanelli: Istituto Zooprofilattico Sperimentale della Sicilia "A. Mirri", Via Marinuzzi, 90129 Palermo, Italy, alessandra.stancanelli@izssicilia.it

Alexander Tavella: Istituto Zooprofilattico Sperimentale delle Venezie, 35020 Legnaro, PD, Italy, atavella@izsvenezie.it

Giuliana Terracciano: Istituto Zooprofilattico Sperimentale del Lazio e della Toscana "M. Aleandri", 00178 Rome, Italy, giuliana.terracciano@izslt.it

Calogero Terregino: Istituto Zooprofilattico Sperimentale delle Venezie, 35020 Legnaro, PD, Italy, cterregino@izsvenezie.it

Matteo Tonni: Istituto Zooprofilattico Sperimentale della Lombardia e dell'Emilia Romagna "Bruno Ubertini" (IZSLER), Via Antonio Bianchi 7/9, 25124 Brescia, Italy, matteo.tonni@izsler.it

Jacopo Zema: Istituto Zooprofilattico Sperimentale dell'Umbria e delle Marche "Togo Rosati", Via Salvemini 1, 06126 Perugia, Italy, j.zema@izsum.it

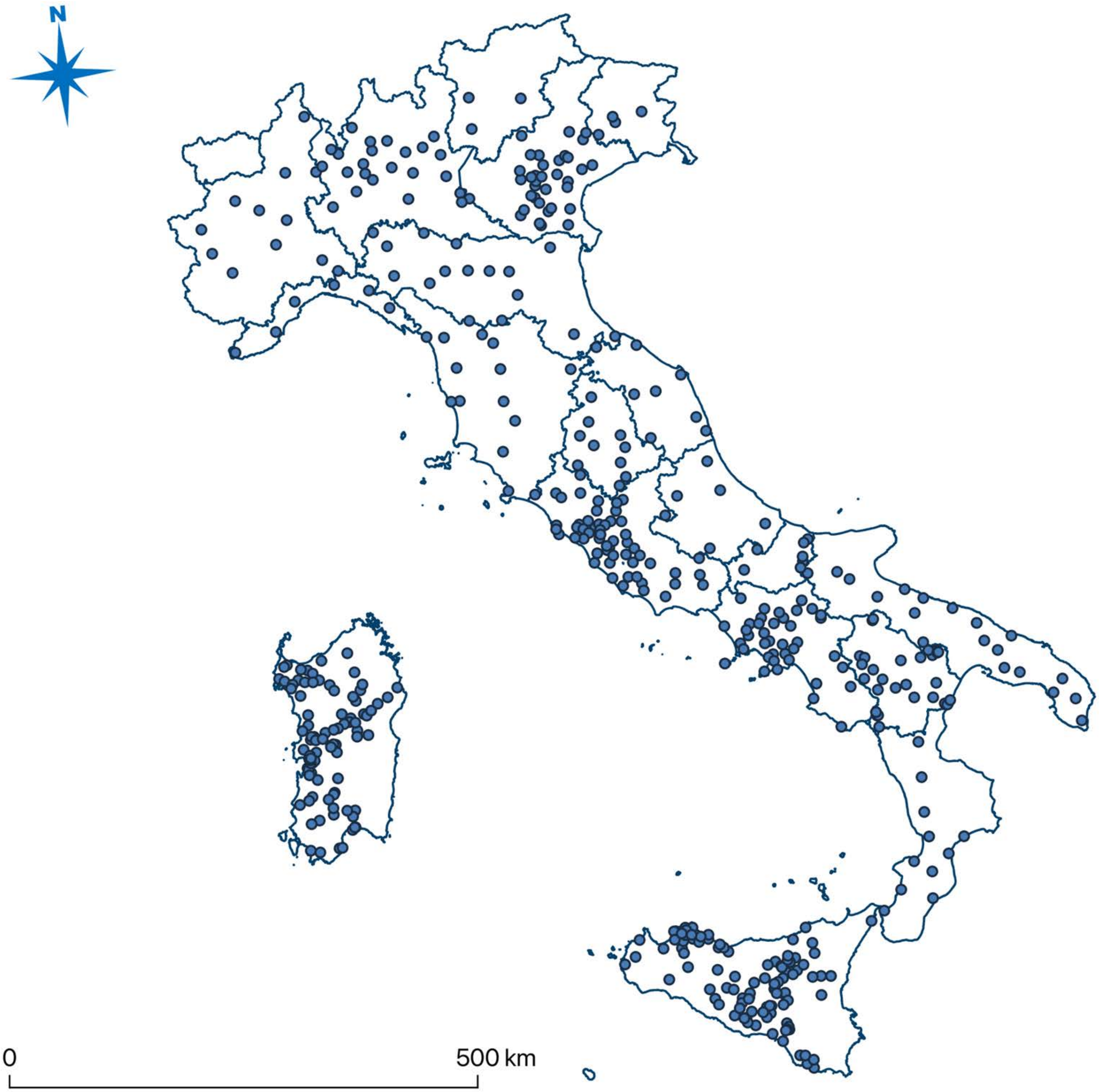

Figures

Figure S1. Collection sites (blue dots) of the serum samples belonging to the Equestrian (EQ) category tested for EqHV by RT Real-Time PCR. Only Regional borders are indicated.

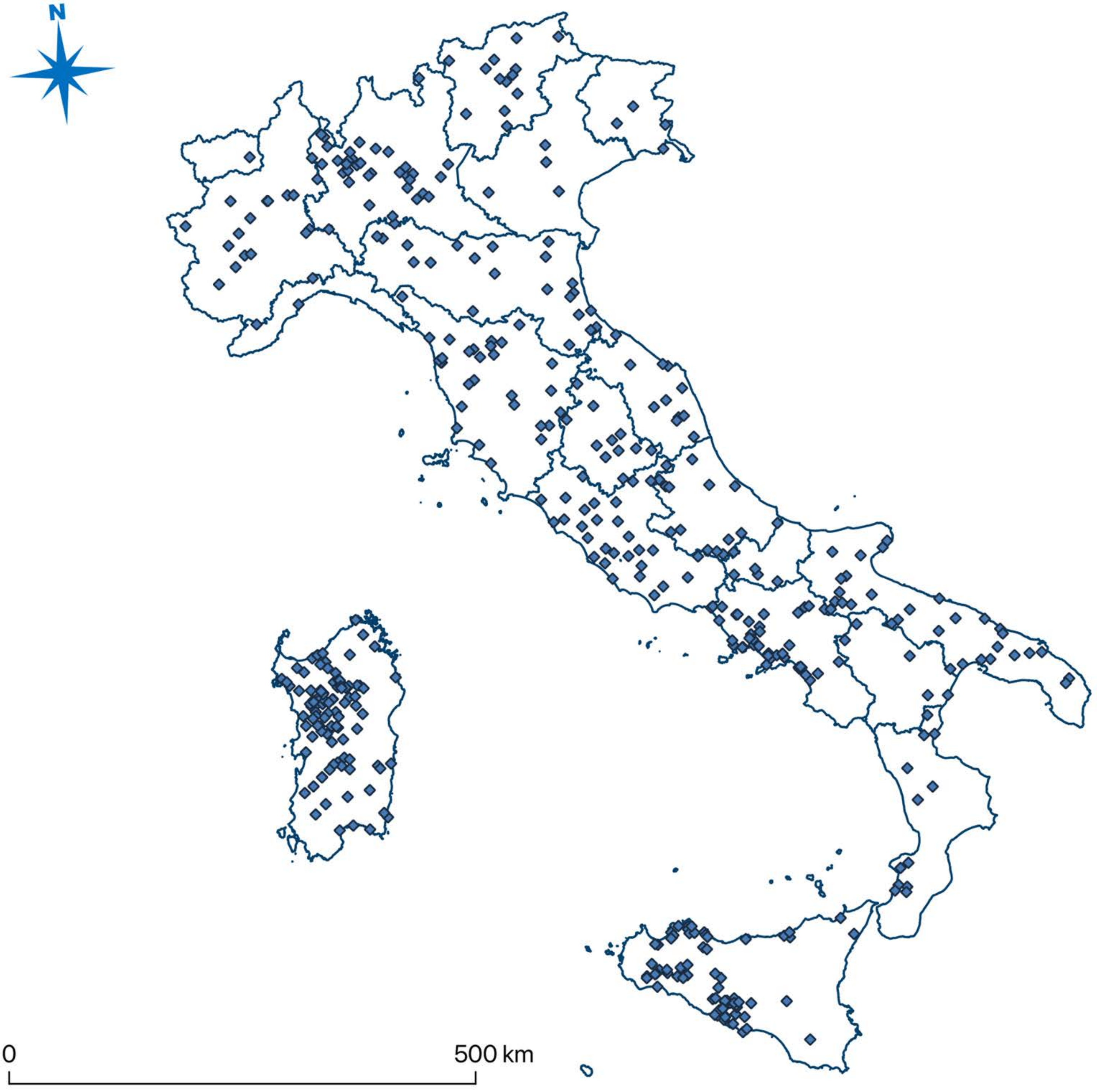

Figure S2. Collection sites (blue diamonds) of the serum samples belonging to the Competition (COMP) category tested for EqHV by RT Real-Time PCR. Only Regional borders are indicated.

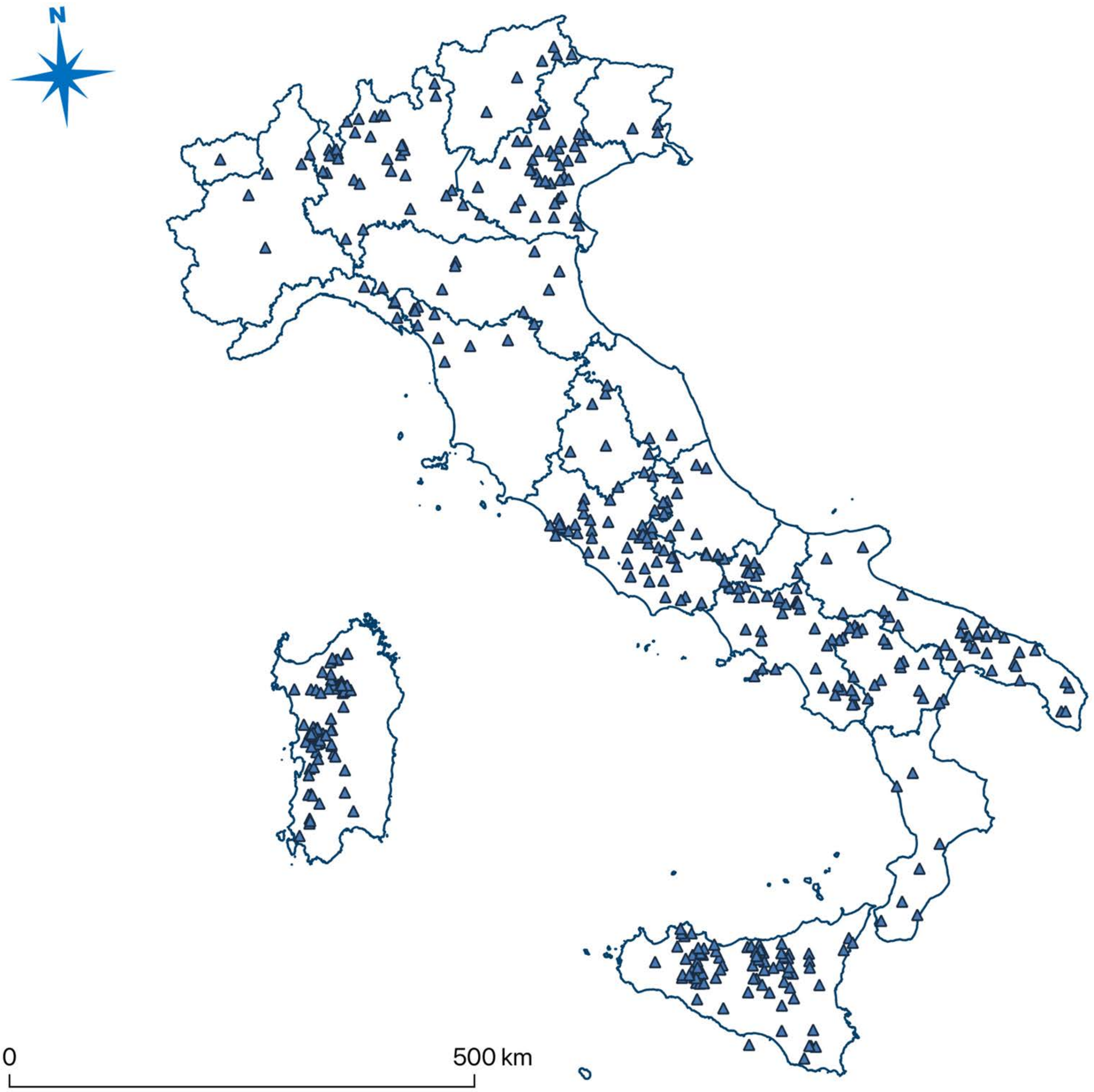

Figure S3 Collection sites (blue triangles) of the serum samples belonging to the Work/Meat (W/M) category tested for EqHV by RT Real-Time PCR. Only regional borders are indicated.

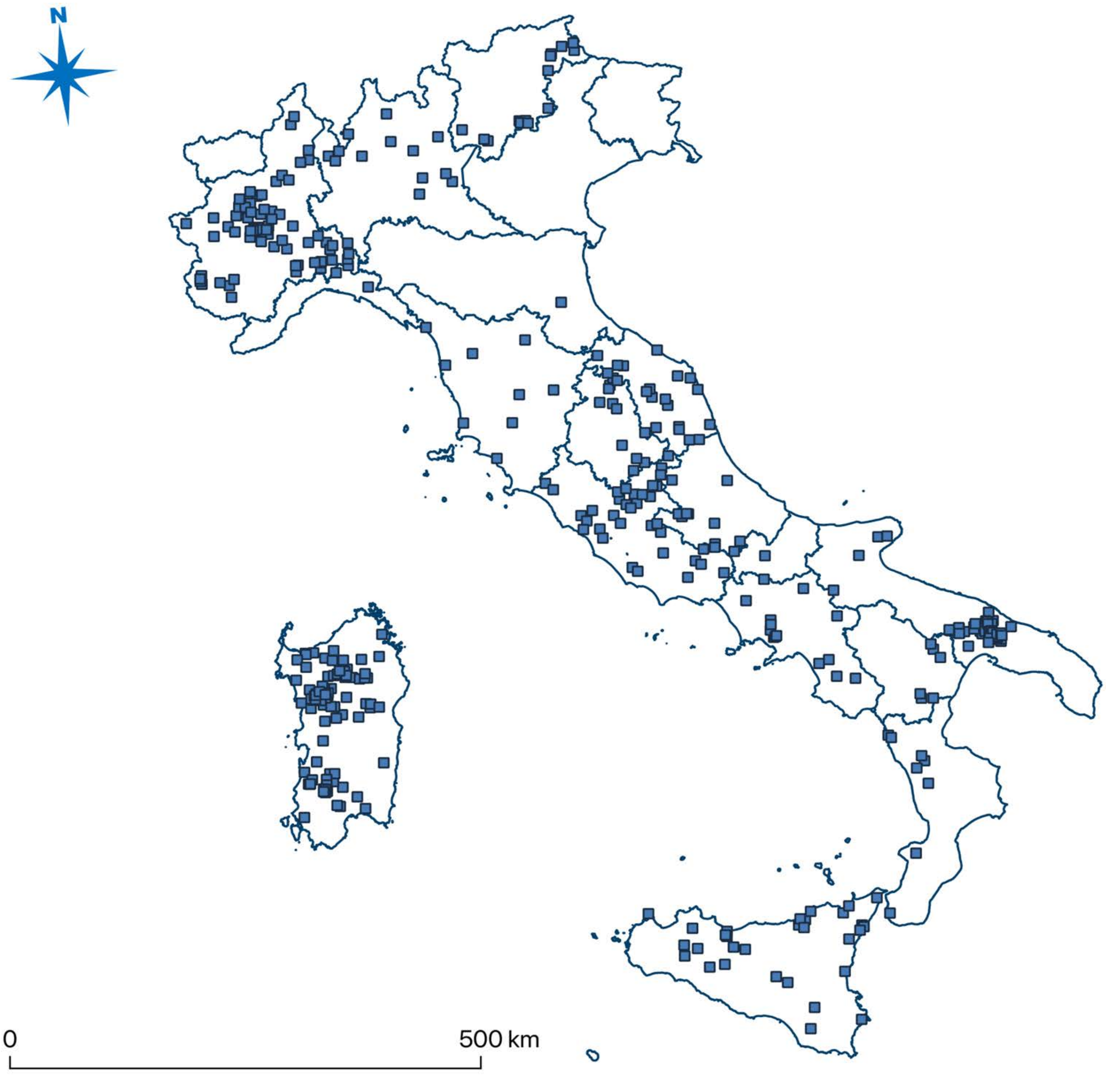

Figure S4 Collection sites (blue squares) of the serum samples belonging to the Reproduction (REP) category tested for EqHV by RT Real-Time PCR. Only regional borders are indicated.

Tables

Table S1. Primers and probe for RT-Real time PCR [2] and primers for Endpoint PCR [3] respectively used for the amplification of the 5-UTR fragment and NS3 of the EqHV genome.

	ID	Sequence 5′-3′	Target	Type	Ref.
Primers	Qanti-5UF1	GAGGGAGCTGRAATTCGTGAA	5′UTR	Real-time RT-PCR	[2]
Primers	Qanti-5UR1	GCAAGCATCCTATCAGACCGT	5′UTR	Real-time RT-PCR	[2]
Probe	[BHQ1a]-FAM	CCACGAAGGAAGGCGGGGGC	5′UTR	Real-time RT-PCR	[2]
Primers	equHepaci-4397-Fw	GTTCCTCATCCTAACATCGA	NS3	Conventional RT-PCR	[3]
Primers	equHepaci-5006-Rv	TCTCCCAAAACTCAGTATGGT C	NS3	Conventional RT-PCR	[3]
